# Author Correction: Engineered bacterial voltage-gated sodium channel platform for cardiac gene therapy

**DOI:** 10.1038/s41467-023-43260-9

**Published:** 2023-11-16

**Authors:** Hung X. Nguyen, Tianyu Wu, Daniel Needs, Hengtao Zhang, Robin M. Perelli, Sophia DeLuca, Rachel Yang, Michael Pan, Andrew P. Landstrom, Craig Henriquez, Nenad Bursac

**Affiliations:** 1 Department of Biomedical Engineering, Duke University, Durham, NC USA; 2 Department of Pediatrics, Division of Cardiology, Duke University School of Medicine, Durham, NC USA; 3 Department of Cell Biology, Duke University School of Medicine, Durham, NC USA

**Keywords:** Cardiology, Molecular medicine, Gene therapy

Correction to: *Nature Communications* 10.1038/s41467-022-28251-6, published online 02 February 2022

Since the publication of this work, Michael Pan has changed their name from Michael Tian. This has now been amended in the HTML and PDF versions of the article.

