# Supplementary material for: Engineered bacterial voltage-gated sodium channel platform for cardiac gene therapy
Source: Nat Commun. 2022 Feb 2;13:620. doi: 10.1038/s41467-022-28251-6 (PMC8810800; doi:10.1038/s41467-022-28251-6)
Supplement: Supplementary file 1 — Supplementary information [file 41467_2022_28251_MOESM1_ESM.pdf]

Supplementary Figures

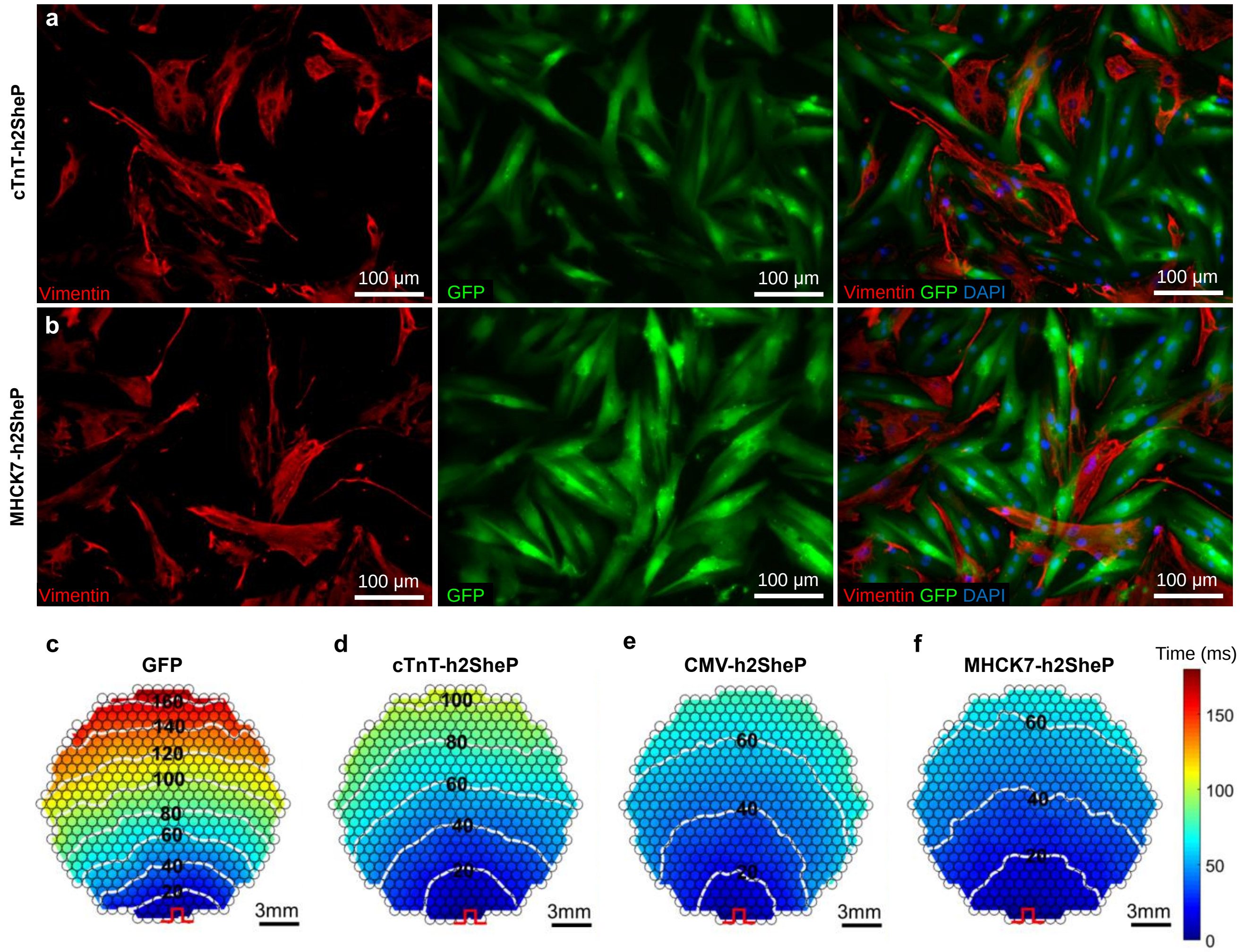

Supplementary Fig. 1: Optimized BacNa$_v$ expression improves cardiac AP conduction in NRVM monolayers. a,**b** Representative images of cocultured NRVM-fibroblast monolayers transduced with cTnT-h2SheP-2A-GFP (**a**) and MHCK7-h2SheP-2A-GFP (**b**) lentiviruses showing that Vimentin$^+$ fibroblasts did not express GFP. **c**-**f**, Representative isochrone activation maps of optically recorded AP conduction in cocultured NRVM monolayers transduced with lentiviruses expressing GFP (**c**), cTnT-h2SheP-2A-GFP (**d**), CMV-h2SheP-2A-GFP (**e**), and MHCK7-h2SheP-2A-GFP (**f**). Pulse signs indicate locations of stimulating electrodes. Circles denote 504 recording sites. Results of quantitative analysis of AP conduction are shown in Fig. 2.

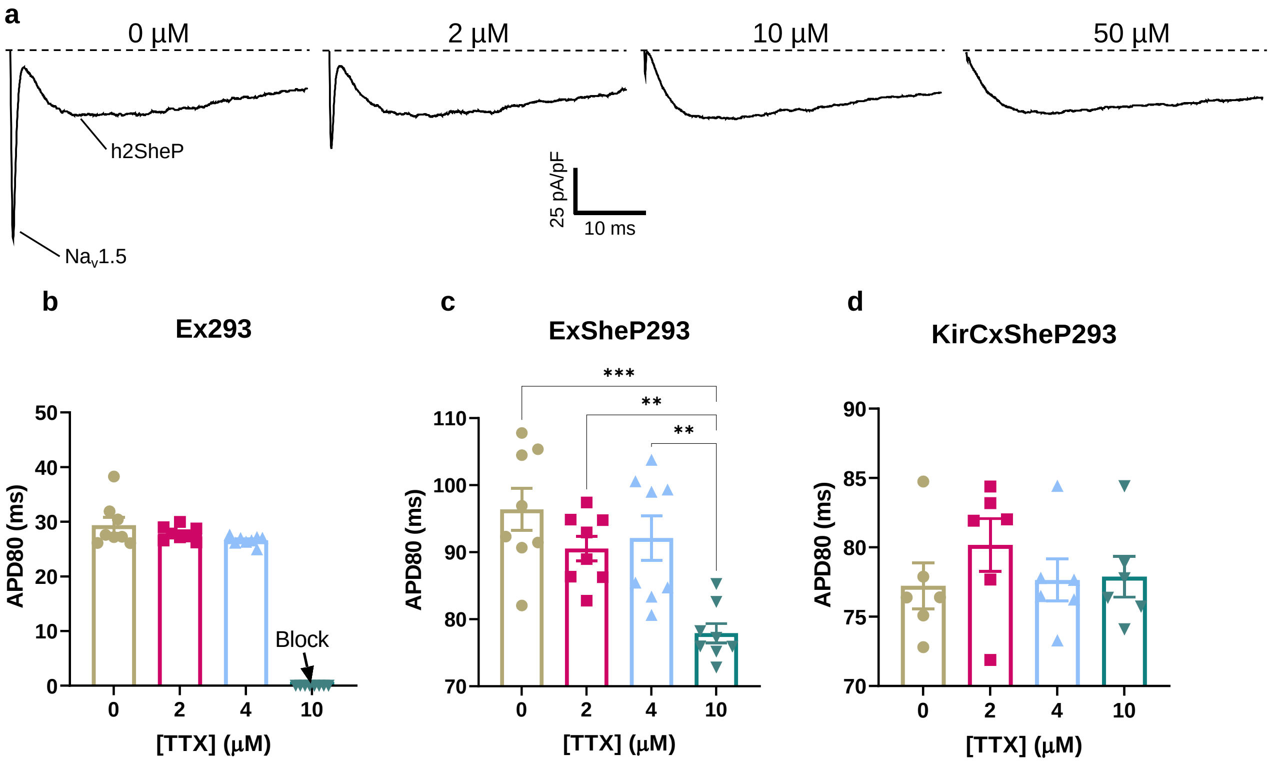

Supplementary Fig. 2: TTX inhibits endogenous Na$_v$1.5 but not BacNa$_v$ current in engineered HEK293 cells. a, Representative voltage-clamp recordings after stepping membrane potential from -80 mV (holding potential) to 0 mV in ExSheP293 cells expressing K$_{ir}$2.1, Cx43, Na$_v$1.5, and h2SheP, showing reduced Na$_v$1.5 current with increasing TTX concentrations but no change in h2SheP current. Dashed line denotes zero current level. **b-d**, Effects of increasing TTX concentrations on APD$_{80}$ of Ex293 (**b**, n = 8; expressing K$_{ir}$2.1, Cx43, Na$_v$1.5), ExSheP293 (**c**, n = 8), and KirCxSheP293 (**d**, n = 6; expressing K$_{ir}$2.1, Cx43, and h2SheP) cell lines (for other parameters see Fig. 3). **P=0.0028, 4 µM vs. 10 µM group; **P=0.0085, 2 µM vs. 10 µM group; ***P=0.0001, 0 µM vs. 10 µM group in **c**. Error bars indicate s.e.m; statistical significance was determined by one-way ANOVA, followed by Tukey's post-hoc test to calculate P values. Source data are provided as a Source Data file.

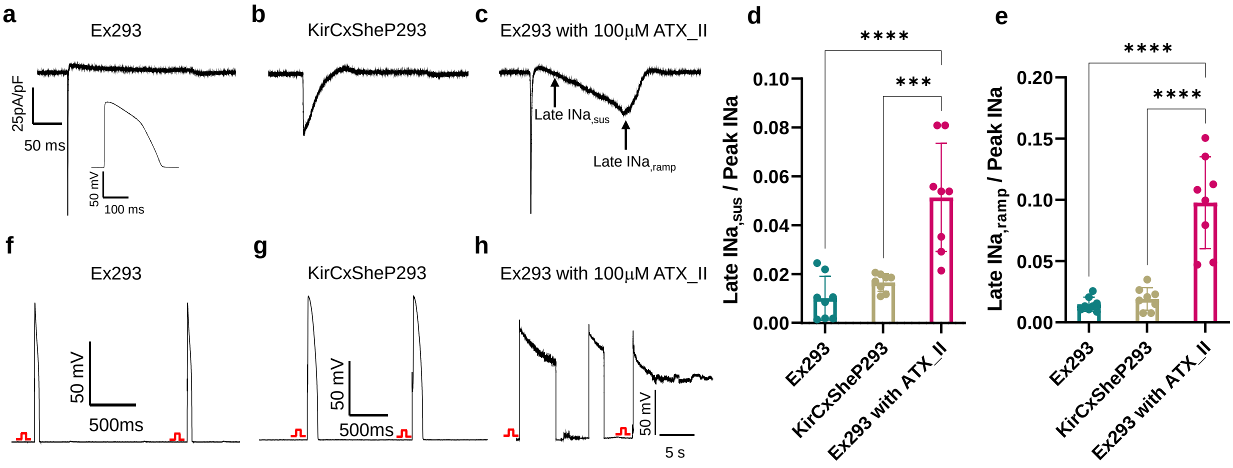

Supplementary Fig. 3: BacNa$_v$ current is distinct from ATX II induced late Nav1.5 current. a-c, Representative Na current (I_{Na}) traces recorded during application of a modeled human AP in the AP-clamp, voltage-clamp mode in Na$_v$1.5-expressing Ex293 cells (**a**), BacNa$_v$-expressing KirCxSheP293 cells (**b**), and Ex293 cells treated with 100 µm ATX II (**c**). Recordings were performed at 25^{0}C in presence of 1mM $BaCl_2$. **d**,**e** Corresponding quantifications from **a-c**) of late $I_{Na,sus}$ measured at 100ms after the onset of AP (**d**) and late $I_{Na,ramp}$ measured as maximum late I_{Na} during AP repolarization (**e**) ***P=0.0002; ****P<0.0001. n=8 per group. **f-h,** Representative AP traces recorded via current clamp in Ex293 cells (**f**), KirCxSheP293 cells (**g**) and Ex293 cells treated with 100 µm ATX II (**h**). Red pulse signs indicate time of current stimulation. Error bars indicate s.e.m; statistical significance in **d,e** was determined by one-way ANOVA, followed by Tukey's post-hoc test to calculate P values. Source data are provided as a Source Data file.

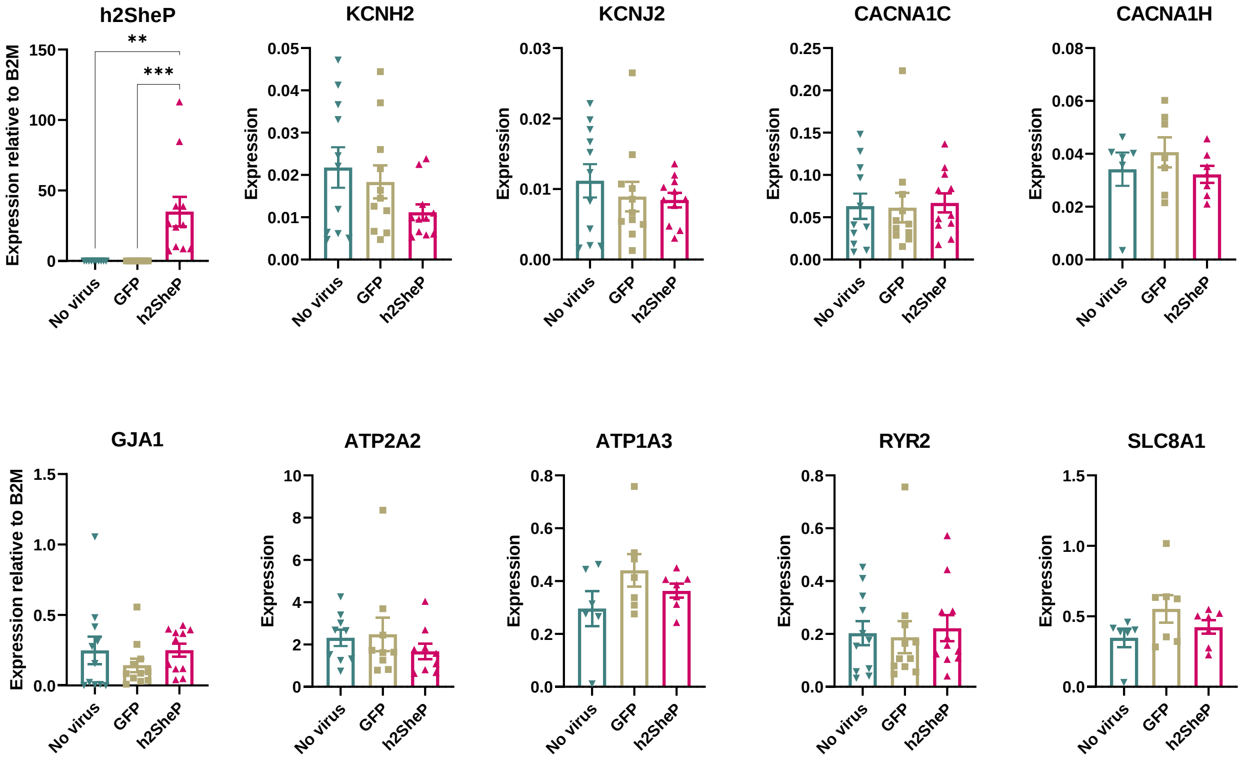

Supplementary Fig. 4: Stable expression of BacNa$_V$ in NRVMs does not alter expression of endogenous cardiac ion channel and transporter genes. All mRNA expression levels for specified genes are normalized to expression level of B2M housekeeping gene. n=7 biologically independent samples for ATP1A3, CACNA1H and SLC8A1 expression, n=9 biologically independent samples for ATP2A2 expression and n=11 biologically independent samples for h2SheP, KCNH2, KCNJ2, RYR2, CACNA1C and GJA1 expression. **P=0.0011, ***P=0.0009 vs. h2SheP group for h2SheP expression. Error bars indicate s.e.m; statistical significance was determined by one-way ANOVA, followed by Tukey's post-hoc test to calculate P values. Source data are provided as a Source Data file.

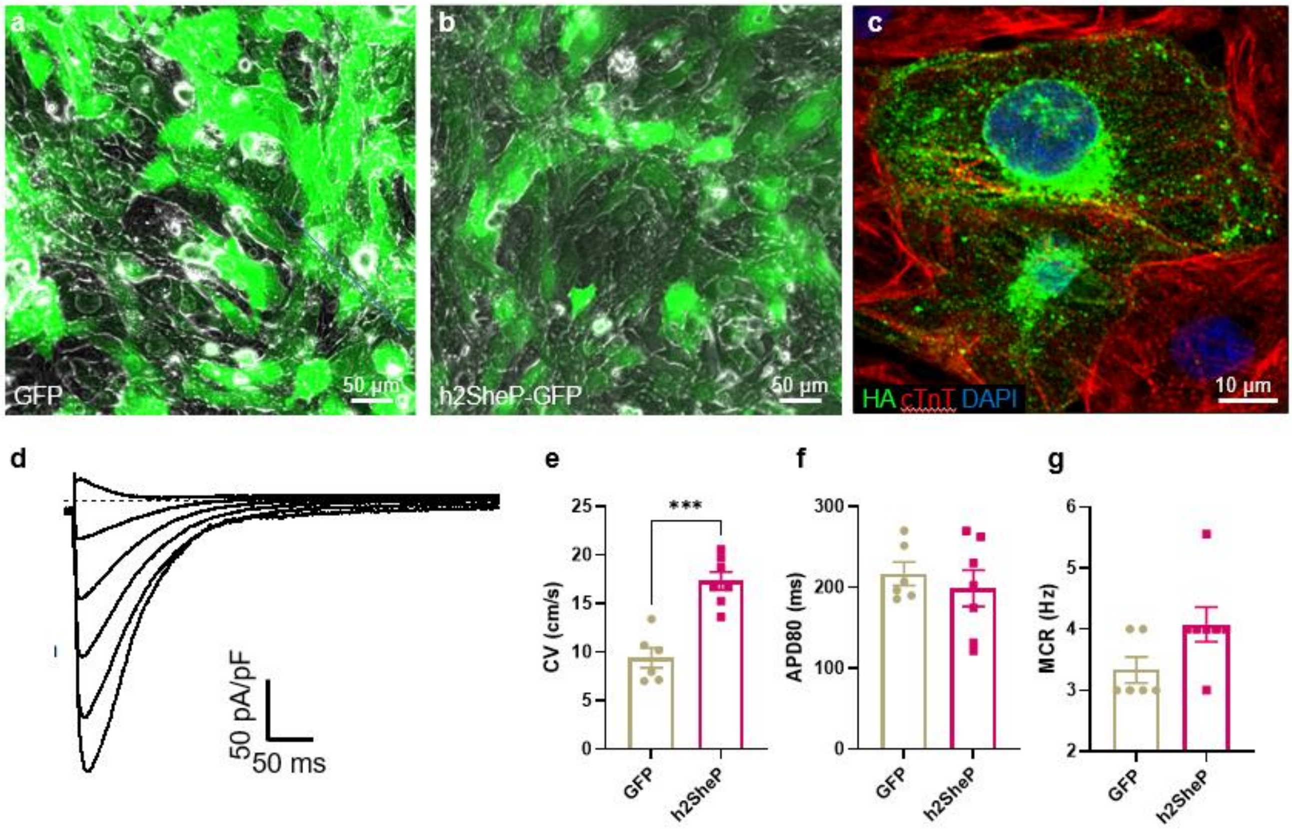

Supplementary Fig. 5: BacNa$_v$ expression improves AP conduction in hiPSC-CM monolayers. **a**,**b**, Representative images of hiPSC-CMs transduced with CMV-GFP (**a**) or MHCK7-h2SheP-2A-GFP (**b**) lentiviruses. **c,** Representative image of hiPSC-CMs transduced with CMV-h2SheP-HA lentivirus showing efficient channel trafficking to membrane. **d**, Representative voltage-clamp recordings of sodium current in response to steps of membrane voltages from -80 mV (holding potential) to -10 to +40 mV in hiPSC-CMs transduced with CMV-h2SheP-HA lentivirus. Slow kinetics of Na current inactivation demonstrates the existence of robust BacNa$_v$ current. **e-g**, Quantifications of CV (**e**), APD$_{80}$ (**f**) and MCR (**g**) during AP propagation optically recorded in hiPSC-CM monolayers transduced with MHCK7-h2SheP-2A-GFP ("h2SheP", n = 7) or control MHCK7-GFP ("GFP", n = 6) lentivirus. ***P=0.0001 versus GFP group in **e**. Error bars indicate s.e.m; statistical significance was determined by an unpaired two-tailed Student's t-test to calculate P values. Source data are provided as a Source Data file.

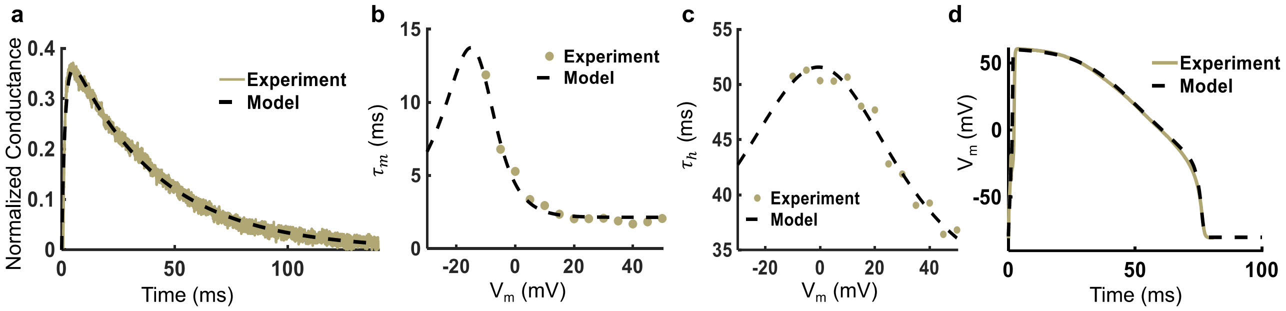

Supplementary Fig. 6: Optimized computational model of BacNa$_v$ channel shows excellent agreement with experimental results. a, Representative experimental and simulated traces of normalized h2SheP conductance during voltage-clamp step from -80mV to -10 mV. **b**,**c**, Time constants of activation (**b**) and inactivation (**c**) extracted from experimental data and corresponding curves generated from the simulations using optimized BacNa$_v$ model. **d**, Representative patch-clamp AP recording of HEK293 cell expressing K$_{ir}$2.1 and h2SheP and corresponding model-fit trace. Note excellent agreement between experimental and simulated data.

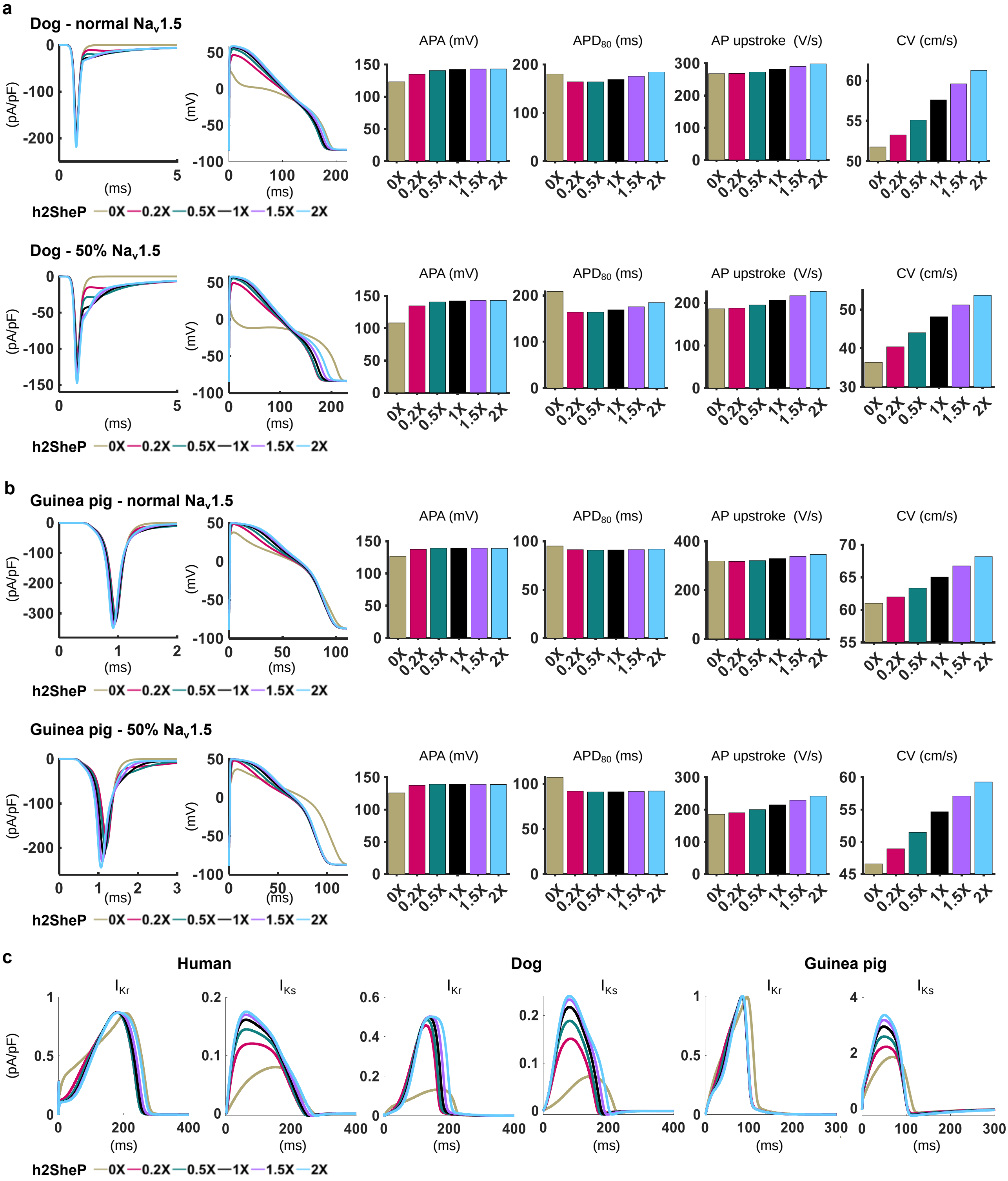

Supplementary Fig. 7: Simulated h2SheP expression results in increased excitability, improved AP conduction, and augmented I_{Ks} in computational models of adult ventricular myocytes. a,**b**, Simulated effects of various h2SheP expression levels (0X to 2X) on total sodium current, AP shape, amplitude (APA), duration (APD_{80}), and maximum upstroke velocity (AP upstroke), and conduction velocity (CV) in adult dog (**a**) and guinea pig (**b**) ventricular myocyte models under normal and reduced (50% of normal $Na_v1.5$ current) excitability. **c**, Simulated effects of various h2SheP expression levels (0X to 2X) on I_{Kr} and I_{Ks} currents shown during human, dog, and guinea pig ventricular myocyte APs.

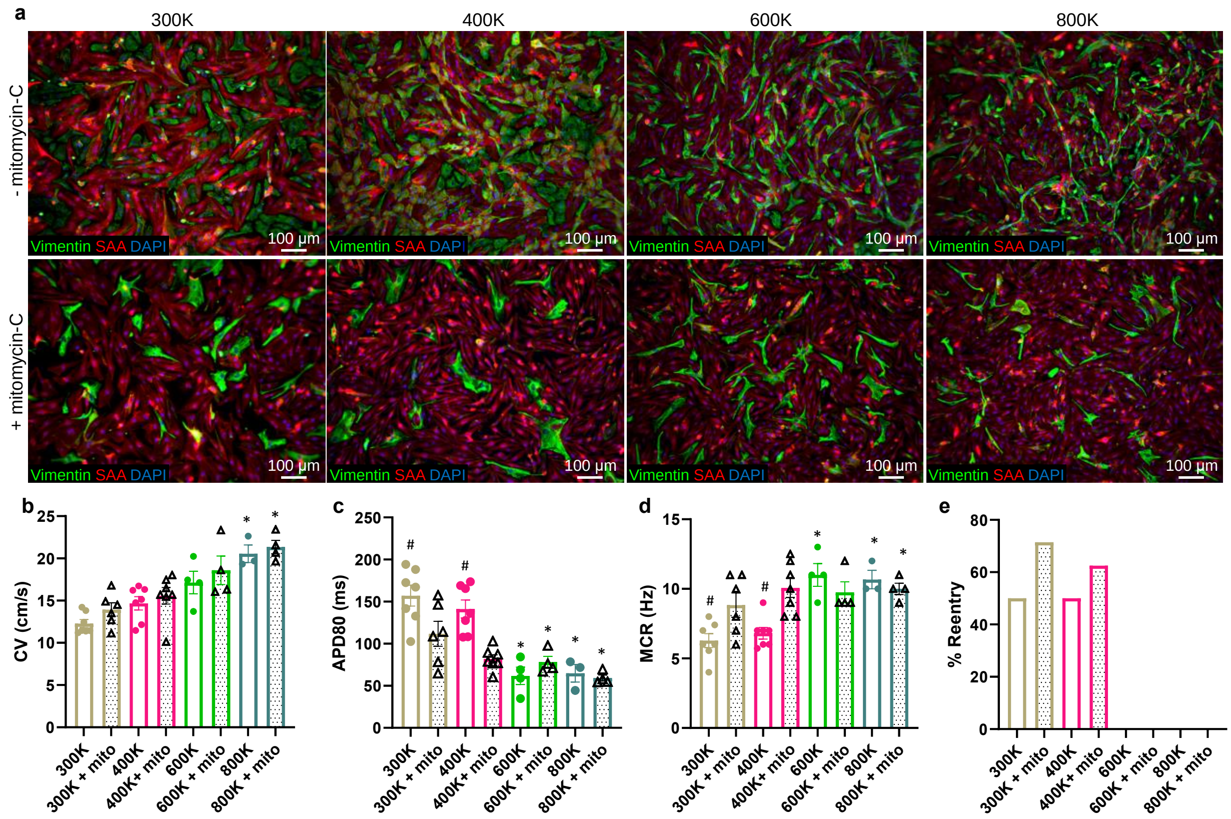

Supplementary Fig. 8: Varying cell seeding density and treatment with mitomycin-C yield highly arrhythmogenic NRVM cultures. a, Representative immunostaining images of NRVM cultures with varying cell seeding densities (from 300-800K cells per well of a 12-well plate), with and without mitomycin-C treatment. Increasing cell seeding density led to a higher content of vimentin^{+} fibroblasts at intermediate densities (400K) and changes in fibroblast morphology at higher densities (600K and 800K), while mitomycin-C treatment effectively reduced fibroblast content in all conditions. **b-d**, Effects of cell seeding density and mitomycin-C treatment (+mito) on CV (**b**), APD_{80} (**c**), and MCR (**d**) of NRVM monolayers (n = 3, 800k; n = 4, 600k, 600k+mito, 800k+mito; n = 6, 300k+mito; n = 7, 300k, 400k, 400k+mito). **e**, Incidence of reentry in NRVM cultures was significantly more prevalent at lower cell seeding densities and further increased with mitomycin-C treatment. *P<0.05 from both 300K and 400K groups and #P<0.05 vs. corresponding +mito group. Exact P values for all comparisons are included in **Source Data**. Error bars indicate s.e.m; statistical significance was determined by two-way ANOVA, followed by Tukey's post-hoc test to calculate P values. Source data are provided as a Source Data file.

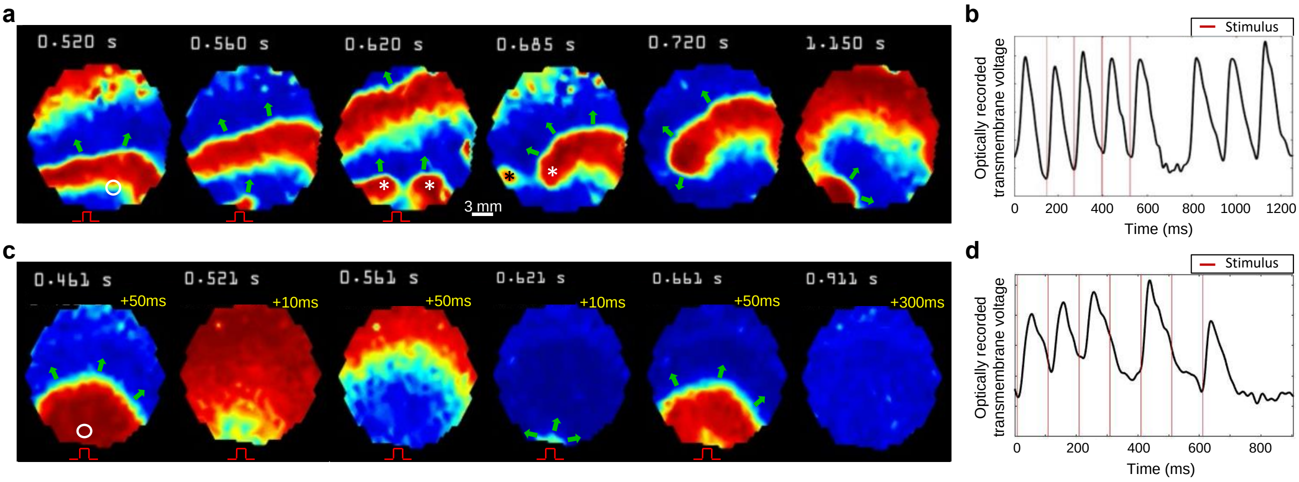

Supplementary Fig. 9: BacNa$_V$ expression in arrhythmogenic NRVM monolayers prevents induction of reentrant activity. a, Representative movie snapshots of optically recorded membrane potentials in arrhythmogenic NRVM monolayers transduced with control MHCK7-GFP lentivirus, shown during rapid point pacing which yielded reentry induction (see also Supplementary Movie 3). Rapid pacing (8 Hz) from the bottom of the monolayer generated a wave break and two transiently coexisting waves (white asterisks) distal to the pacing site, one which was annihilated against the tissue boundary (black asterisk) and another which continued to stably rotate after pacing was terminated. **b,** Representative membrane voltage trace optically recorded from the specified monolayer site (white circle) in **a**, showing the pacing-induced (red lines) action potentials, followed by self-sustained activity due to reentry. **c,** Representative snapshots of optically recorded membrane voltage shown during rapid pacing in arrhythmogenic NRVM monolayers transduced with MHCK7-h2SheP-2A-GFP lentivirus (see also Supplementary Movie 3). Rapid pacing (10 Hz) from the bottom of the monolayer did not result in the formation of distal wave break, but rather induced a proximal conduction block at the pacing site, every other beat (1:2 conduction). In **a,c,** elapsed time (in seconds) since the beginning of recording is shown in white font above the voltage snapshot. Blue and red color in snapshots indicate resting and activated tissue, respectively. Green arrows denote directions of the propagating wave fronts. Pulse signs indicate locations of stimulus electrode. The lack of pulse sign denotes that pacing was terminated. Numbers in ms in **c** specify time elapsed from the last paced stimulus; the stimulus delivered at 0.511s did not elicit AP propagation (as evident from no new launched wave at 0.521s and 0.561s snapshots). **d,** Representative membrane voltage trace optically recorded from the specified monolayer site (white circle) in **c**, showing the initial 1:1 tissue capture, followed by the partial capture of every other pacing-induced AP, and eventually cessation of all activity after pacing is terminated, thus not resulting in reentry induction. In **b,d,** red lines denote times when pacing stimuli were delivered.

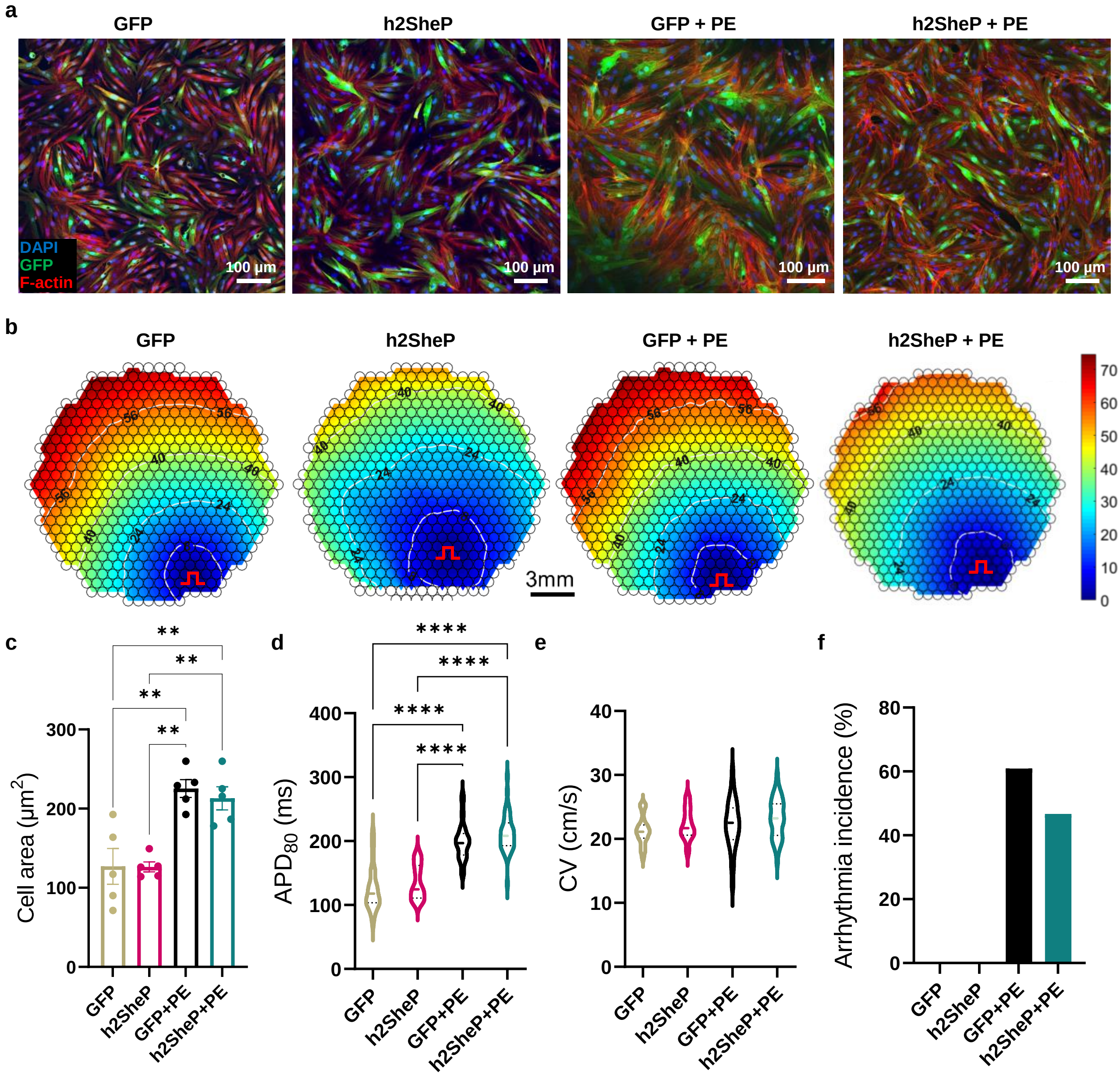

Supplementary Fig. 10: BacNa$_V$ expression in phenylephrine-treated NRVM monolayers does not decrease incidence of triggered activity. a, Representative immunostaining images of NRVM monolayers transduced with control MHCK7-GFP ("GFP") or MHCK7-h2SheP-2A-GFP ("h2SheP") lentivirus, showing larger cell size in the phenylephrine (PE) treated groups. **b**, Representative isochrone activation maps of AP propagation in non-treated or PE-treated ("+PE") NRVM monolayers transduced with specified lentivirus. Pulse signs indicate location of pacing electrode and circles denote 504 recording sites. **c-e**, Cell area (**c**, n=5, **P=0.0013, GFP vs. GFP+PE; **P=0.0044, GFP vs. h2SheP+PE; **P=0.0013, h2SheP vs. GFP+PE; **P=0.0041, h2SheP vs. h2SheP+PE), AP duration (APD_{80}, **d,** ****P<0.0001), conduction velocity (CV, **e**), and incidence of triggered activity (**f**, see also Supplementary Movie 4) in the four monolayer groups. Phenylephrine treatment in GFP+PE (n=46) and h2SheP+PE (n=45) monolayers resulted in increased cell area, APD80, and arrhythmia incidence, but not CV, compared to untreated GFP (n = 24) or h2SheP (n = 24) monolayers. h2SheP expression did not significantly decrease incidence of triggered activity induced by PE (P=0.1742). Error bars indicate s.e.m; statistical significance in **c-e** was determined by one-way ANOVA, followed by Tukey's post-hoc test to calculate P values; statistical significance in **f** was determined by two-sided Chi square test. Source data are provided as a Source Data file.

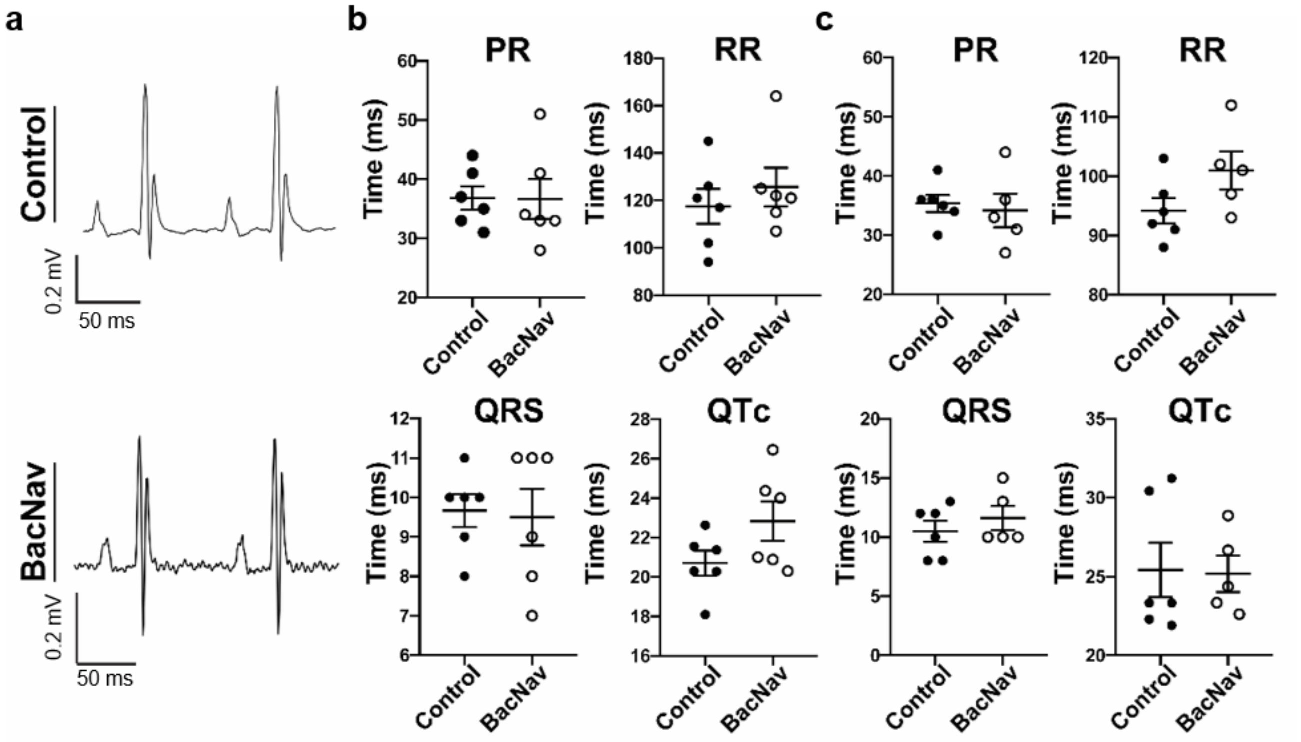

Supplementary Fig. 11: Intravenous AAV-mediated delivery of BacNa$_V$ does not alter cardiac electrophysiology in healthy mice. a, Representative surface ECG traces in mice six weeks after injection of $1x10^{12}$ vg of scAAV-MHCK7-GFP (Control) or scAAV-MHCK7-h2SheP-HA (BacNa$_V$). **b-c**, ECG parameters in control (n=6) and BacNa$_V$ (n=5) groups measured before (**b**) and after (**c**) administration of 200µg/g caffeine and 1µg/g isoproterenol. Error bars indicate s.e.m. PR, PR-interval duration; RR, RR-interval duration; QRS, QRS-complex duration; QTc, corrected QT-interval duration. Source data are provided as a Source Data file.

Supplementary Table 1: List of qPCR primers. All rat primers (B2M as house-keeping gene) were used for studies of NRVM genes. All human primers (GAPDH as house-keeping gene) were used for studies of HEK293 genes.

Gene	Description	Species	Forward Primer	Reverse Primer
ATP1A3	Na+/K+ ATPase alpha 3	Rat	CTGTCATCTTCCTCATCGGTATC	GACAGTTCTTCCGAGCCAT
ATP2A2	SERCA-2a	Rat	TGTGCTCTGTGTAATGACTCTG	CTCCGTGTCGAATACATTCATCT
B2M	Beta-2 Microglobulin	Rat	GCTTGCAGAGTTAAACACGTC	CCAGATGATTCAGAGCTCCAT
CACNA1C	L-type Ca Channel (Ca_v1.2)	Rat	ACTTCATCATCCTCTTCATCTGTG	CCAGCTTCTTTCTCTCCTTCTC
CACNA1H	T-type Ca Channel (Ca_v3.2)	Rat	CACTCGTTCTACAACTTCATCTACT	CTCTGAGAACTGTGTGGCTATC
GAPDH	Glyceraldehyde-3-phosphate dehydrogenase	Human	AAGGTGAAGGTCGGAGTCAA	ATGAAGGGGTCATTGATGG
GJA1	Gap junction protein α-1 (Connexin 43)	Rat	GAGCTGTCGATTATGGAGGA	AGGTTCAGTTGGGGGATG
h2SheP	Bacteria voltage-gated Na channel	Rat	CCTTACGCCTGGGTGTTCTT	CAGCGCATGTTCTTCATCGG
KCNH2	Voltage-gated inwardly rectifying potassium channel (K_v11.1)	Rat	CAGTGACCGGGAAATCATAGC	ACATTGTGGGTCCGCTCTTT
KCNJ2	Inward-rectifier potassium channel (K_{ir}2.1)	Rat	GTGTCTGAGGTCAACAGCTT	GAAACCATAGCCGATGGTTG
KCNJ2	Inward-rectifier potassium channel (K_{ir}2.1)	Human	TGTCACGGATGAATGCCCAA	CTGCGCCAATGATGAAAGCA
RYR2	Ryanodine receptor 2	Rat	GCAGTTGATTGAGCCTAGTGT	CCTCTTTGACTTCCAGTTTAGAGT
SCN5A	Voltage-gated Na channel (Na_v1.5)	Rat	CGTAACTTCACCGAGCTCAA	CCCACATAGTAACACATCCGT
SCN5A	Voltage-gated Na channel (Na_v1.5)	Human	TTCAGGGCTGAAGACCATCG	GCACTTGTGCCTTAGGTTGC
SLC8A1	Sodium/calcium exchanger	Rat	CCTATAAAACCATTGAAGGCACAG	TTTTCTCATACTCCTCGTCATCG

Supplementary Methods:

Wild-type and codon-optimized sequences of Na_vSheP D60A

<u>bSheP:</u>

ATGAGTACATCTTTACTTAACGCGCCAACGGGTTTGCAGGCACGAGTGATTAACTTGGT
TGAGCAAAACTGGTTTGGTCATTTTATTTTGGCATTGATTTTAATCAACGCGGTGCAGTT
AGGTATGGAGACCTCAGCCAGCCTGATGGCGCAATACGGTACTTTGTTGATGAGTCTTG
ATAAGTTGCTACTGAGTGTATTTGTGGTGGAGTTATTGCTGCGGATTTATGCCTACAGGG
GGAAATTTTTTAAAGACCCTTGGAGCGTGTTCGATTTTACCGTGATAGTGATAGCACTGA
TCCCTGCATCTGGGCCATTGGCTGTCCTGCGTTCGCTCAGGGTATTGCGGGTGCTGAGAG
TGTTAACAATTGTGCCATCAATGAAACGGGTGGTGTCTGCGCTGTTGGGATCACTTCCTG
GATTGGCATCGATCGCCACGGTATTACTGTTGATTTATTATGTGTTTGCGGTGATTGCTA
CCAAAATTTTTGGCGATGCATTCCCTGAATGGTTTGGCACTATTGCTGACTCATTTTATA
CCCTATTTCAAATAATGACGCTTGAAAGCTGGTCTATGGGAATTTCGCGGCCAGTGATG
GAAGTGTACCCTTATGCTTGGGTATTTTTCGTACCATTTATTCTGGTAGCGACTTTCACA
ATGCTAAATTTGTTTATTGCGATTATCGTCAATACCATGCAAACCTTCAGCGACGAAGA
GCATGCATTAGAGCGTGAACAAGACAAACAAATCTTAGAGCAGGAACAAAGACAAATG
CACGAGGAGTTGAAAGCCATCAGACTCGAGCTACAACAATTACAAACCTTGTTGCGCAA
TGCTGCTGGTGATTCTTCTAATGTGTCGACAAAGGGAAACATTGGTTCTGACTGA

<u>hSheP:</u>

ATGTCAACCTCACTGCTGAACGCTCCAACTGGGCTGCAGGCAAGAGTCATCAATCTGGT
CGAACAGAACTGGTTTGGGCACTTTATTCTGGCACTGATCCTGATTAACGCAGTGCAGC
TGGGAATGGAGACCAGCGCCTCCCTGATGGCACAGTACGGAACACTGCTGATGTCCCTG
GCAAAGCTGCTGCTGAGCGTGTTCGTGGTCGAACTGCTGCTGCGAATCTACGCCTATCG
GGGCAAGTTCTTTAAAGACCCCTGGAGCGTGTTCGACTTCACCGTGATCGTCATTGCCCT
GATTCCAGCTAGTGGACCTCTGGCCGTGCTGCGGTCACTGAGAGTGCTGAGGGTCCTGC
GCGTGCTGACAATCGTGCCTAGCATGAAGAGGGTGGTCTCAGCTCTGCTGGGCAGCCTG
CCAGGACTGGCATCCATCGCTACTGTGCTGCTGCTGATCTACTATGTCTTCGCAGTGATC
GCCACTAAAATTTTCGGAGACGCTTTTCCCGAGTGGTTCGGCACCATCGCAGATTCTTTT
TATACACTGTTCCAGATCATGACTCTGGAGTCTTGGAGTATGGGCATCAGTCGCCCAGT
CATGGAAGTGTACCCCTATGCCTGGGTCTTCTTTGTGCCTTTTATTCTGGTCGCCACCTTC
ACAATGCTGAACCTGTTTATCGCTATCATTGTGAATACTATGCAGACCTTTAGCGACGA
GGAACACGCTCTGGAGCGAGAACAGGATAAGCAGATTCTGGAGCAGGAACAGAGACA
GATGCATGAGGAACTGAAAGCAATCAGGCTGGAGCTGCAGCAGCTGCAGACACTGCTG
AGAAACGCTGCTGGCGATTCATCAAACGTGTCCACTAAAGGAAACATTGGCTCTGACTG
A

<u>h2SheP:</u>

ATGTCAACCTCCCTTCTGAACGCCCCCACCGGTCTGCAAGCCCGCGTCATCAACCTGGTC
GAACAGAACTGGTTCGGCCACTTCATCCTCGCACTGATTCTCATTAACGCCGTGCAGCTT
GGAATGGAAACTAGCGCGTCCCTGATGGCTCAATACGGCACACTGCTCATGAGCCTGGC
GAAGCTGCTCCTGTCCGTGTTCGTGGTGGAACTGTTGCTGCGGATCTATGCGTACCGCG
GAAAATTCTTCAAGGATCCATGGAGCGTGTTCGACTTTACTGTGATTGTGATCGCACTCA
TCCCGGCCTCGGGACCGCTCGCCGTGCTCCGGTCACTGAGAGTCCTGAGGGTGCTCAGA
GTGCTGACCATTGTGCCTAGCATGAAGCGCGTGGTGTCCGCCCTGTTGGGATCCCTGCC
GGGTTTGGCTTCGATTGCCACTGTGCTGCTCCTGATCTACTACGTGTTCGCCGTCATTGC
CACTAAGATTTTCGGCGACGCCTTTCCTGAGTGGTTCGGAACCATCGCTGACTCTTTCTA
CACCTTGTTCCAAATCATGACCCTGGAATCCTGGTCCATGGGGATTTCGAGGCCCGTGA
TGGAGGTGTACCCTTACGCCTGGGTGTTCTTCGTCCCCTTCATCCTTGTCGCAACCTTCA
CCATGCTTAACCTGTTTATCGCCATCATCGTGAACACGATGCAGACCTTCTCCGATGAAG
AACATGCGCTGGAGCGGGAACAGGACAAGCAGATCCTGGAGCAGGAACAGCGGCAGA
TGCACGAGGAGCTGAAGGCCATCCGGCTGGAGCTGCAGCAGCTCCAAACTCTGCTGCGC
AACGCGGCCGGAGATTCAAGCAATGTGTCGACCAAGGGGAACATCGGCTCCGACTGA

MATLAB code for computational modeling of Na$_v$SheP D60A

This function takes the current membrane voltage (v), maximum BacNa$_v$ conductivity (GNa), an output switch, and the current values for the gating state variables (m and h). If the output switch is 1, output is the steady-state conductivity (gna), gating state variables (minf, hinf), and the time constants for that specific voltage value. If the output switch is 2, output is the conductivity (gna), and derivatives for the state variables (dm, dh). If used in a cell model, gna should be multiplied by the driving force (V-E_{Na}) to get BacNa$_v$ current. dm and dh can be used directly with ode15s or other solvers, or multiplied by dt in an explicit Euler solver.

```
function [gna, varargout] = bacnav_channel_paper(v, GNa, outswitch, varargin)
if outswitch==2
    if nargin==5
        m = varargin(1);
        h = varargin(2);
    else
        error('Please enter the correct number of parameters for mh');
    end
end

minf_v50_mhleg = -28.34;
minf_slope_mhleg = 5.33;
hinf_v50_mhleg = -77.21;
hinf_slope_mhleg = 8.32;
taum_mhleg_a = 86.37;
taum_mhleg_b = -82.74;
taum_mhleg_c = 17.64;
taum_mhleg_d = -6.008;
taum_mhleg_e = 3.337;
taum_mhleg_f = .4844;
tauh_mhleg_a = 96.17;
tauh_mhleg_b = 10.45;
tauh_mhleg_c = -23.26;
tauh_mhleg_d = 2.529;
minf = sigmoid(v, minf_v50_mhleg, minf_slope_mhleg);
hinf = 1-sigmoid(v, hinf_v50_mhleg, hinf_slope_mhleg);
taum = hypsec(v, taum_mhleg_a, taum_mhleg_b, taum_mhleg_c, taum_mhleg_d, taum_mhleg_e,
taum_mhleg_f);
tauh = sigmoid_gen(v, tauh_mhleg_a, tauh_mhleg_b, tauh_mhleg_c, tauh_mhleg_d);

if outswitch==2
    gna =  GNa .* m .* h;
elseif outswitch==1
    gna = GNa .* minf .* hinf;
end

if outswitch==2
    dm = (minf-m)./taum;
    dh = (hinf-h)./tauh;
    varargout{1} = dm;
    varargout{2} = dh;
elseif outswitch==1
    varargout{1} = minf;
```

```
    varargout{2} = hinf;
    varargout{3} = taum;
    varargout{4} = tauh;
end
end

function val = hypsec(v, a, b, c, d, e, f)
val = a./(exp((v - b)/c) + exp(-(v - d)/e)) + f;
end

function val = gaussian(v, a, b, c, d)
val = a.*exp(-((v-b)/c).^2)+ d;
end

function val = sigmoid(v, minf_v50, minf_slope)
val = 1./(1+exp((minf_v50-v)./minf_slope));
end

function val = sigmoid_sum(v, a, b, c, d, e, f, g, h)
val = a+(b-a)*sigmoid(v,c,d) + f + (e-f)*sigmoid(v,g,h);
end

function val = sigmoid_gauss(v, a, b, c, d, e, f, g, h)
val = a + (b-a)*sigmoid(v,c,d) + gaussian(v,e,f,g,h);
end

function val = sigmoid_gen(v, a, b, c, d)
val = (a-(a-b)./(1+exp((c-v)/d)));
end
```
