# Supplementary material for: Engineered bacterial voltage-gated sodium channel platform for cardiac gene therapy
Source: Nat Commun. 2022 Feb 2;13:620. doi: 10.1038/s41467-022-28251-6 (PMC8810800; doi:10.1038/s41467-022-28251-6)
Supplement: Supplementary file 3 — Description of Additional Supplementary Files [file 41467_2022_28251_MOESM3_ESM.docx]

**Description of Additional Supplementary Files**

File Name: Supplementary Movie 1

Description: Simulation of AP propagation in a model of human ventricular tissue with 15% non-conducting obstacles.

File Name: Supplementary Movie 2

Description: Simulation of AP propagation in a model of human ventricular tissue with 20% of anisotropic non-conducting obstacles.

File Name: Supplementary Movie 3

Description: Effect of rapid pacing in arrhythmogenic NRVM monolayers. Pulse signs indicate location of pacing electrode.

File Name: Supplementary Movie 4

Description: Effect of phenylephrine on NRVM monolayers. Pulse signs indicate location of pacing electrode. Pacing pulses are denoted with white flashes. Representative membrane voltage trace shown was optically recorded at location distal to the pacing site (white asterisk).
