# Supplementary material for: Engineered bacterial voltage-gated sodium channel platform for cardiac gene therapy
Source: Nat Commun. 2022 Feb 2;13:620. doi: 10.1038/s41467-022-28251-6 (PMC8810800; doi:10.1038/s41467-022-28251-6)
Supplement: Supplementary file 8 — Reporting Summary [file 41467_2022_28251_MOESM8_ESM.pdf]

nature portfolio

Corresponding author(s): Nenad Bursac

Last updated by author(s): Dec 16, 2021

Reporting Summary

Nature Portfolio wishes to improve the reproducibility of the work that we publish. This form provides structure for consistency and transparency in reporting. For further information on Nature Portfolio policies, see our Editorial Policies and the Editorial Policy Checklist.

Statistics

For all statistical analyses, confirm that the following items are present in the figure legend, table legend, main text, or Methods section.

n/a	Confirmed	
☐	☒	The exact sample size (*n*) for each experimental group/condition, given as a discrete number and unit of measurement
☐	☒	A statement on whether measurements were taken from distinct samples or whether the same sample was measured repeatedly
☐	☒	The statistical test(s) used AND whether they are one- or two-sided<br>*Only common tests should be described solely by name; describe more complex techniques in the Methods section.*
☒	☐	A description of all covariates tested
☐	☒	A description of any assumptions or corrections, such as tests of normality and adjustment for multiple comparisons
☐	☒	A full description of the statistical parameters including central tendency (e.g. means) or other basic estimates (e.g. regression coefficient) AND variation (e.g. standard deviation) or associated estimates of uncertainty (e.g. confidence intervals)
☐	☒	For null hypothesis testing, the test statistic (e.g. *F*, *t*, *r*) with confidence intervals, effect sizes, degrees of freedom and *P* value noted<br>*Give P values as exact values whenever suitable.*
☒	☐	For Bayesian analysis, information on the choice of priors and Markov chain Monte Carlo settings
☒	☐	For hierarchical and complex designs, identification of the appropriate level for tests and full reporting of outcomes
☒	☐	Estimates of effect sizes (e.g. Cohen's *d*, Pearson's *r*), indicating how they were calculated

Our web collection on statistics for biologists contains articles on many of the points above.

Software and code

Policy information about availability of computer code

Data collection	Optical mapping: LabView 8.5<br>Patch clamp: WinWCP 5.1.6, MultiClamp 700B Commander<br>Imaging: Andor Fusion 2.2<br>Computational modeling: MATLAB R2015b<br>Electrocardiogram: LabScribe v4
Data analysis	Statistical analysis: Graphpad Prism 9.02<br>Image analysis and visualization: ImageJ 1.52<br>Optical mapping analysis: MATLAB R2015b<br>Patch clamp analysis: WinWCP 5.1.6, MATLAB R2015b<br>Flow cytometry: FlowJo v10.7.1<br>Human codon optimization: OptimumGene algorithm (Genscript); ATUM Gene-GPS™ algorithm (DNA2.0)

For manuscripts utilizing custom algorithms or software that are central to the research but not yet described in published literature, software must be made available to editors and reviewers. We strongly encourage code deposition in a community repository (e.g. GitHub). See the Nature Portfolio guidelines for submitting code & software for further information.

Data

Policy information about availability of data

All manuscripts must include a data availability statement. This statement should provide the following information, where applicable:

- Accession codes, unique identifiers, or web links for publicly available datasets
- A description of any restrictions on data availability
- For clinical datasets or third party data, please ensure that the statement adheres to our policy

The following statement has been added in the data availability section: "All other data generated and/or analyzed are available within the manuscript and its supplementary information files. Source data are provided as a Source Data file."

Field-specific reporting

Please select the one below that is the best fit for your research. If you are not sure, read the appropriate sections before making your selection.

[x] Life sciences [ ] Behavioural & social sciences [ ] Ecological, evolutionary & environmental sciences

For a reference copy of the document with all sections, see nature.com/documents/nr-reporting-summary-flat.pdf

Life sciences study design

All studies must disclose on these points even when the disclosure is negative.

Sample size	Sample size for murine experiment was based on a t-test to test for differences in ECG parameters. A study with an effect size of 0.25 and a power of 80% required a minimum of n = 5 independent biological replicates per group, to test this difference at 5% significance using a two-tailed test.
Data exclusions	No data was excluded in this study.
Replication	Replications are mentioned in figure legends and the Statistics analysis section of the Methods. Technical and experimental replicates performed are noted in figure legends. Experiments were repeated a minimum of 3 times using biologically independent specimens (in vivo studies) or 3 times in technically independent replicates (in vitro studies). All attempts at replication produced similar results.
Randomization	For all murine experiments, mice were randomized to different treatment groups prior to experimental treatment.<br>For all none murine in vitro experiments, all cell culture wells were randomly allocated into different experimental and treatment groups prior to any drug treatment and viral transduction.
Blinding	For ECG measurements, all investigators were blinded for data collection and analysis.<br>For AAV injections, the animal surgeon was blinded to treatment allocations. Single CM isolation, tissue processing and image acquisition were performed by individuals blinded to group allotment.

Reporting for specific materials, systems and methods

We require information from authors about some types of materials, experimental systems and methods used in many studies. Here, indicate whether each material, system or method listed is relevant to your study. If you are not sure if a list item applies to your research, read the appropriate section before selecting a response.

Materials & experimental systems

n/a	Involved in the study	
[ ]	[x]	Antibodies
[ ]	[x]	Eukaryotic cell lines
[x]	[ ]	Palaeontology and archaeology
[ ]	[x]	Animals and other organisms
[x]	[ ]	Human research participants
[x]	[ ]	Clinical data
[x]	[ ]	Dual use research of concern

Methods

n/a	Involved in the study	
[x]	[ ]	ChIP-seq
[x]	[ ]	Flow cytometry
[x]	[ ]	MRI-based neuroimaging

Antibodies

Antibodies used	Primary antibody:<br>anti-sarcomeric α-actinin (Sigma, a7811)<br>anti-vimentin (Abcam, ab92547)<br>anti-Cx43 (LSBio, LS-B9770)

	anti-HCN4 (Alomone, APC-052)<br>anti-cardiac troponin T (Sigma, ab45932)<br>anti-HA tag (Cell Signaling Technology, C29F4)<br>Secondary antibody:<br>Chicken anti-Mouse IgG (H+L) Cross-Adsorbed Secondary Antibody, Alexa Fluor 488 (Thermo Fisher Scientific, A-21200/A-21441)<br>Chicken anti-Mouse IgG (H+L) Cross-Adsorbed Secondary Antibody, Alexa Fluor 594 (Thermo Fisher Scientific, A-21201/A-21442)<br>Chicken anti-Mouse IgG (H+L) Cross-Adsorbed Secondary Antibody, Alexa Fluor 647 (Thermo Fisher Scientific, A-21463)<br>Donkey anti-Rabbit IgG (H+L) Highly Cross-Adsorbed Secondary Antibody, Alexa Fluor Plus 594 (Thermo Fisher Scientific, A-32754)<br>Donkey anti-Rabbit IgG (H+L) Highly Cross-Adsorbed Secondary Antibody, Alexa Fluor Plus 647 (Thermo Fisher Scientific, A-32795)<br>Alexa Fluor 488-conjugated phalloidin (Thermo Fisher Scientific, A12379)<br>Alexa Fluor 647-conjugated phalloidin (Thermo Fisher Scientific, A22287)<br>DAPI (Sigma, D9542)
Validation	Primary antibody:<br>anti-sarcomeric α-actinin (Sigma, a7811): Validation was stated on the manufacturer's website and confirmed for IHC and WB using human and rat muscle. Species reactivity: fish, snake, frog, goat, hamster, pig, canine, mouse, feline, chicken, lizard, bovine, human, sheep, rat, rabbit. Suitable for immunohistochemistry, ELISA, immunofluorescence and western blot.<br>anti-vimentin (Abcam, ab92547): Validation was stated on the manufacturer's website and confirmed for IF, IHC and WB using human, mouse and rat samples and knockout-validation. Species reactivity: Mouse, Rat, Human, African green monkey. Suitable for: Flow Cyt (Intra), ICC/IF, WB, IHC-P.<br>anti-Cx43 (LSBio, LS-B9770): Validation was stated on the manufacturer's website and validated for IF, IHC and WB. Confirmed for IHC using human samples. Species reactivity: Rat, Human, Monkey, Mouse, Dog (tested or 100% immunogen sequence identity). Suitable for IHC, IHC-P, IF, WB.<br>anti-HCN4 (Alomone, APC-052): Validation was stated on the manufacturer's website. Knockout validation in mouse heart. Species reactivity: Mouse, Rat, Human. Suitable for ICC, IF, IHC, IP, WB.<br>anti-cardiac troponin T (Abcam, ab45932): Validation was stated on the manufacturer's website and tested for IHC, ELISA and WB. Species reactivity: Human. Suitable for IHC, WB.<br>anti-HA tag (Cell Signaling Technology, C29F4): Validation was stated on the manufacturer's website. Validated using SimpleChIP® Enzymatic Chromatin IP Kits. Species reactivity: All species expected. Suitable for W, IP, IHC, ChIP. IF, F, E-P.<br>Secondary antibody:<br>Alexa Fluor 488 (Thermo Fisher Scientific, A-21200/A-21441): Validation was stated on the manufacturer's website. Suitable for: Immunohistochemistry (IHC), Immunocytochemistry (ICC/IF).<br>Alexa Fluor 594 (Thermo Fisher Scientific, A-21201/A-21442): Validation was stated on the manufacturer's website. Suitable for: Immunohistochemistry (IHC), Immunocytochemistry (ICC/IF).<br>Alexa Fluor 647 (Thermo Fisher Scientific, A-21463): Validation was stated on the manufacturer's website. Suitable for: Immunohistochemistry (IHC), Immunocytochemistry (ICC/IF).<br>Alexa Fluor 488-conjugated phalloidin (Thermo Fisher Scientific, A12379): Validation was stated on the manufacturer's website. Suitable for: Immunohistochemistry (IHC), Immunocytochemistry (ICC/IF).<br>Alexa Fluor 647-conjugated phalloidin (Thermo Fisher Scientific, A22287): Validation was stated on the manufacturer's website. Suitable for: Immunohistochemistry (IHC), Immunocytochemistry (ICC/IF).<br>DAPI (Sigma, 1:300): Validation was stated on the manufacturer's website. Suitable for fluorescence.

Eukaryotic cell lines

Policy information about cell lines

Cell line source(s)	HEK293T (ATCC, CRL-3216)<br>HEK293 (ATCC, CRL-1573)<br>hiPSCs were reprogrammed from BJ fibroblasts (ATCC cell line, CRL-2522) at the Duke University iPSC Core Facility and named DU11 (Duke University clone #11)
Authentication	HEK 293, HEK293T cells were authenticated by vendors by STR profiling.<br>hiPSCs were authenticated by standard tests, including pluripotency testing with pluripotency marker expression using IF and FACS, karotyping to confirm genomic integrity, and teratoma formation.
Mycoplasma contamination	Mycoplasma testing was performed routinely in the lab. All cell lines tested negative.
Commonly misidentified lines<br>(See ICLAC register)	No commonly misidentified lines were used in this study

Animals and other organisms

Policy information about studies involving animals; ARRIVE guidelines recommended for reporting animal research

Laboratory animals	Male 6 –10 week old CD-1 mice (Charles River Laboratories, Wilmington MA)<br>Male and female 2-day-old Sprague-Dawley rats (Charles River Laboratories, Wilmington MA)<br>All mice were housed in 12-hour light/dark cycles, at ambient temperatures of 70-74 degrees Fahrenheit, at a humidity range between 30-70%, and with access to food and water ad libitum, in accordance with animal study guidelines at Duke University.
Wild animals	No wild animals were used in this study

Field-collected samples	No field-collected samples were used in this study
Ethics oversight	All animal studies were performed in accordance with the animal protocol A064-21-03 approved by the Duke University Institutional Animal Care and Use Committee.

Note that full information on the approval of the study protocol must also be provided in the manuscript.
